# Comparative Analysis of State Fish Consumption Advisories Targeting Sensitive Populations

**DOI:** 10.1289/ehp.11372

**Published:** 2008-08-01

**Authors:** Alison C. Scherer, Ami Tsuchiya, Lisa R. Younglove, Thomas M. Burbacher, Elaine M. Faustman

**Affiliations:** 1 Department of Environmental and Occupational Health Sciences, School of Public Health and Community Medicine, University of Washington, Seattle, Washington, USA; 2 Institute for Risk Analysis and Risk Communication, University of Washington, Seattle, Washington; 3 Center for Ecogenetics and Environmental Health, University of Washington, Seattle, Washington

**Keywords:** advisory/advisories, benefits, consumption, contaminants, fish, nutrition/nutritional, pregnant women, risks, sensitive populations, women of childbearing age

## Abstract

**Objective:**

Fish consumption advisories are issued to warn the public of possible toxicological threats from consuming certain fish species. Although developing fetuses and children are particularly susceptible to toxicants in fish, fish also contain valuable nutrients. Hence, formulating advice for sensitive populations poses challenges. We conducted a comparative analysis of advisory Web sites issued by states to assess health messages that sensitive populations might access.

**Data sources:**

We evaluated state advisories accessed via the National Listing of Fish Advisories issued by the U.S. Environmental Protection Agency.

**Data extraction:**

We created criteria to evaluate advisory attributes such as risk and benefit message clarity.

**Data synthesis:**

All 48 state advisories issued at the time of this analysis targeted children, 90% (43) targeted pregnant women, and 58% (28) targeted women of childbearing age. Only six advisories addressed single contaminants, while the remainder based advice on 2–12 contaminants. Results revealed that advisories associated a dozen contaminants with specific adverse health effects. Beneficial health effects of any kind were specifically associated only with omega-3 fatty acids found in fish.

**Conclusions:**

These findings highlight the complexity of assessing and communicating information about multiple contaminant exposure from fish consumption. Communication regarding potential health benefits conferred by specific fish nutrients was minimal and focused primarily on omega-3 fatty acids. This overview suggests some lessons learned and highlights a lack of both clarity and consistency in providing the breadth of information that sensitive populations such as pregnant women need to make public health decisions about fish consumption during pregnancy.

According to the U.S. Environmental Protection Agency (EPA), “State, tribal, and local governments protect people from possible risks of eating contaminated fish by monitoring their waters and issuing fish advisories when contaminant levels are unsafe” (U.S. EPA 2007a). Finfish and shellfish (herein referred to as fish) consumption may pose health risks due to various contaminants. In July 2007, the U.S. EPA made available online the 2005/2006 National Listing of Fish Advisories (NLFA), which reflects potential chemical risks only. This represents the most recent update of the site as of this writing. According to the NLFA, 3,852 advisories have been issued by states, the District of Columbia, tribes, and U.S. territories because of chemical contamination (U.S. EPA 2007b).

## Fish consumption risks and benefits

Developing fetuses and children are particularly susceptible to toxicants in fish; thus, pregnant women and women of childbearing age represent sensitive populations that may be at higher risk from fish consumption. Approximately 88% of advisories stem from just a handful of contaminants: mercury, polychlorinated biphenyls (PCBs), chlordane, dioxins, and dichlorodiphenyltrichloroethane (DDT) (U.S. EPA 2007b). Fish consumption can confer both risks and benefits (Budtz-Jorgensen et al. 2007; Hibbeln et al. 2007; Mahaffey 2004; Mahaffey and Schoeny 2007; Ponce et al. 2000), and some studies suggest that benefits may outweigh risks for sensitive populations consuming certain fish species (Nesheim and Yaktine 2007). A 2004 joint advisory by the U.S. EPA and the U.S. Food and Drug Administration (FDA) currently recommends that sensitive populations eat up to 12 ounces per week (two average meals) of fish low in mercury as part of a healthy diet, acknowledging the many nutritional benefits of consuming fish (U.S. EPA 2004).

## What is the message to sensitive populations?

To date, no study has comprehensively assessed the health messages contained in fish consumption advisories issued by states. In this analysis, we employed a comparative methodology to assess health messages contained in advisories that sensitive groups, including pregnant women and women of childbearing age, might access through the NLFA. Our objective was to address the following questions: Viewed comprehensively across states, do fish consumption advisories, which we recognize arise from a regulatory context, also address the public health questions that sensitive populations face? Specifically, do advisories convey risk and benefit information on fish species that is sufficient to provide context for the advice offered? Do they provide clarity for these complex risk issues? Clear advice provides pregnant women and women of child-bearing age with the tools and information they need to make healthy, informed decisions regarding fish consumption to optimize their health and the health of their offspring.

## Methods

### Comparative analysis

**Figure 1:**
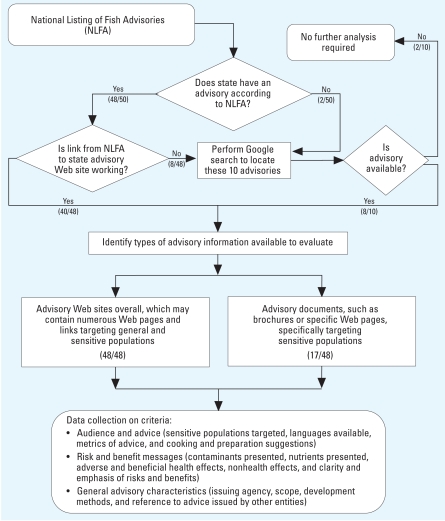
Flow diagram for the comparative analysis of the 48 state fish consumption advisory Web sites assessed.

In this analysis we compared fish consumption advisory information issued by states that we obtained through the NLFA, which represents a means by which pregnant women and women of childbearing age might access fish consumption advice. The analysis represents a snapshot in time—advisories were assessed in either June or July of 2007. Figure 1 is a flow diagram that illustrates the analysis method used. Using the NLFA Contacts page (U.S. EPA 2007c), advisory Web sites were identified for all states that have posted advisories. In instances where Web site links from the NLFA did not work, Google searches were performed to locate advisories because, presumably, this is the next step a pregnant woman or woman of childbearing age might take in search of advisory information. We had no direct contact with officials responsible for issuing advice, because this was an analysis assessing availability as well as context. We identified two types of advisory information for evaluation of selected criteria (Figure 1).

### Criteria assessed

We developed criteria and collected data on advisory attributes shown in Figure 1 regarding audience and advice, risk and benefit messages, and general characteristics. Health effect categories used were adapted from Agency for Toxic Substances and Disease Registry (ATSDR) toxicological profile health effect categories used to present information on hazardous substances (ATSDR 2007). The neurological category was then further broken down into subcategories to reflect the complex nature of references in advisories to these types of health effects. To assess criteria including clarity and emphasis of risk and benefit messages, we developed five-point scales for each of these criteria. We assessed these criteria separately for messages targeting sensitive populations versus the general population. Unless otherwise noted, results are presented for the advisory Web sites overall. Results reflect the authors’ interpretation of criteria developed and applied but do not reflect the interpretations of focus groups of consumers themselves.

## Results

We evaluated 48 Web sites containing fish consumption advice. All but two states, Alaska and Wyoming, had issued fish consumption advice at the time of this analysis. Subsequently, however, both states issued guidelines regarding fish consumption, which highlights the ever- and, at times, quickly evolving nature of this topic. We assessed 40 advisory Web sites located through the NLFA; the Web site addresses linked to advice or to a Web page through which advice could be easily located. We located the remaining eight advisory Web sites via Google searches because the links from the NLFA to state advisory content either did not work [seven cases: Colorado Department of Public Health and Environment, Division of Wildlife (2007), Connecticut Department of Public Health, Environmental Health Section (2006), Delaware Department of Natural Resources and Environmental Control, Department of Health and Social Service’s Division of Public Health (2007), Hawaii State Department of Health (2003), Massachusetts Department of Public Health, Center for Environmental Health–Bureau of Environmental Health Assessment (2007), Nevada State Health Division (2007), and Wisconsin Department of Natural Resources (2007)] or did not provide any apparent fish consumption advice [one case: Utah Department of Health, Office of Epidemiology (2007)].

## Audience and Advice

### Sensitive populations targeted

All Web sites contained at least some advice for sensitive populations, and all but Hawaii (2003) and Nevada (2007) offered advice that was either more strict or more cautiously worded for sensitive populations than for the general population. Advisories issued by Hawaii (2003) and Nevada (2007) addressed only sensitive populations. Seventeen Web sites contained specific brochures or Web pages aimed exclusively at sensitive populations, whereas the rest of the Web sites intermingled advice aimed at sensitive populations with content aimed at members of the general population (e.g., there were no dedicated brochures or Web pages just for sensitive populations). Among the 46 state Web site advisories that offered different advice (either quantitatively or qualitatively different) for sensitive populations, 78% (36) recommended more restrictive meal limits. For instance, the South Dakota Department of Health (2007) advisory recommended less-frequent meals for sensitive populations compared with “healthy adults.” In some cases, consumption frequency recommendations varied across multiple groups. For example, Arizona Game and Fish (2006) offered advice that was progressively less strict for the following categories: children < 6 years of age; women of childbearing age; all other adult women; and men. Several advisories simply recommended that sensitive populations avoid eating contaminated fish under advisory altogether [including the Tennessee Department of Environment and Conservation, Division of Water Pollution Control (2007), Arkansas Department of Health, Game and Fish Commission, Department of Environmental Quality (1999), and South Carolina Department of Health and Environmental Control (2007)]. The Pennsylvania Department of Environmental Protection (2007) and West Virginia Department of Health and Human Resources, Department of Environmental Protection, Division of Natural Resources (2007) advised that sensitive populations be careful about spacing out meals.

Age ranges of the children targeted varied across advisories, from children < 6 years to those < 18 years. Many advisories distinguished between women of childbearing age and nursing women and women who may become or plan to become pregnant (Table 1). “Women of childbearing age” is a blanket category. For example, the North Carolina Department of Public Health (2006) defined women of childbearing age as being between the ages of 15 and 44 years. Several advisories also addressed high-end fish consumers, and Massachusetts (2007), Michigan Department of Community Health, Division of Environmental Health (2007), and Oregon Department of Human Services (2007) addressed those with certain health conditions, such as weak immune systems.

### Languages available

Thirty-eight percent (18) of advisories offered advice in languages in addition to English. Most of these advisories offered advice in one non-English language, but some offered advice in four to six non-English languages [California Office of Environmental Health Hazard Assessment, Fish and Water Quality Evaluation Unit (2003), Vermont Department of Health, Agency of Human Services (2005), and Connecticut (2006)] or even seven non-English languages [Washington State Department of Health (2007) and Massachusetts (2007)]. All advisories that offered non-English advice did so in at least Spanish. Advice was offered in a dozen other languages, as shown in Table 1. Sixty-five percent (11) of the 17 advisory Web sites containing documents specifically for sensitive populations offered advice in non-English languages.

### Metrics of advice: meal frequency and size

All states whose Web sites we reviewed, except Nebraska Department of Health (2007), offered meal frequency advice, given in terms of meals per week, month, year, or a combination thereof (Table 1). In some cases, meal frequency advice was given only in the context of the joint 2004 U.S. EPA/FDA recommendations advising sensitive populations to consume up to 12 ounces per week. The Idaho Department of Health and Welfare (2007) advisory gave meal frequency advice for whole gutted fish versus fillet meals. Seventy-five percent (36) of state Web site advisories gave meal size advice (e.g., in ounces or pounds), while 59% (10) or the 17 advisory documents specifically targeting sensitive populations gave meal size advice. Most states gave advice based on fish length (inches), and some based advice on the size of fish caught [e.g., the North Dakota Department of Health (2005) advisory recommended that sensitive populations not eat certain fish species > 4 pounds].

### Cooking and preparation suggestions

Most advisories gave advice about preparing and cooking fish, such as removing skin and trimming away fat before cooking (Table 1). In addition, most advisories suggested eating smaller, younger fish, which tend to have lower levels of bioaccumulative contaminants, such as mercury. Several states, including Connecticut (2006), Maryland Department of the Environment (2007), Massachusetts (2007), New Hampshire Department of Environmental Services (2007), New Jersey Department of Health and Senior Services, Department of Environmental Protection, Division of Science Research and Technology (2006), and New Mexico Department of Health, Environment Department, Department of Game and Fish, State Parks (2006), warned against eating shellfish hepatopancreas, variously referred to in advisories as crab or lobster tomalley, green gland, mustard, or liver.

## Risk and Benefit Messages

### Contaminants presented

Twenty-six chemical contaminants were responsible for advisories issued by states. Only six advisories addressed single contaminants (only mercury), while the remainder (42) based advice on 2–12 contaminants. In 9 of these 42 multiple-contaminant advisories, the consumption advice was contaminant-specific (e.g., advice for one water body was based on mercury risks whereas another was based on PCB risks) (Table 2). In all but 7 of the 29 cases where advisories did contain advice integrated across contaminants (e.g., advice for a particular water body was based on risks from both mercury and PCBs together), no explanation was given regarding how the integrated advice was developed. In four of the seven instances where some explanation was evident [Delaware (2007), Maryland (2007), Ohio Department of Health, Environmental Protection Agency, Department of Natural Resources (2007), and West Virginia (2007)], the chemical that posed the greatest risk (the “risk driver”) was identified.

Although the NLFA reflects advisories issued because of chemical pollution, there are other contaminants of concern. For example, the New York State Department of Health (2007) advisory recommended anglers harvest only fish that look healthy, because bacteria, viruses, and parasites can cause illness. The Texas Department of State Health Services (2007) advisory noted that shellfish are tested for bacterial contamination. Advisories from California (2003), Washington (2007), and Florida Department of Health (2006) mentioned that they issue shellfish closures because of algal toxins.

### Nutrients presented

Many advisories stated that fish contain nutrients (Table 2). However, 23% did not mention anything about the nutritional value of fish. Seventy-seven percent (37) of advisories mentioned that fish is a source of protein, and 46% (22) mentioned that fish contain omega-3 fatty acids—or beneficial or good oils or fats (which likely refer to omega-3 fatty acids—and which will be discussed in that context). Specifically, 15 advisories mentioned omega-3 fatty acids explicitly, while five mentioned “fish oils,” one mentioned “good fats,” and one mentioned “fatty acids” found in fish. Eleven states referenced other protein sources in addition to fish. For example, advisories issued by the Illinois Department of Health, Fish Contaminant Monitoring Program (2007), Montana Department of Public Health and Human Services, Communicable Disease Control and Prevention Bureau, Food and Consumer Safety Section (2005), and Wisconsin (2007) indicated that fish consumption confers benefits when replacing consumption of high-fat protein sources.

### Adverse and beneficial health effects

**Figure 2:**
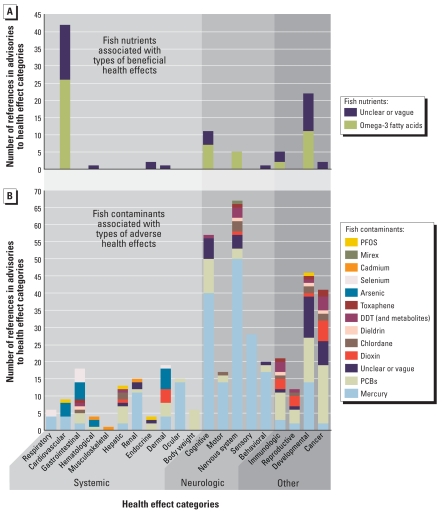
(*A*) Fish nutrients associated with beneficial health effects and (*B*) fish contaminants associated with adverse health effects in state fish consumption advisories. “Unclear or vague” refers to instances where either no nutrient or contaminant was mentioned or the reference was inexact. Advisory references to good or beneficial fats or oils presumably refer to omega-3 fatty acids and are included in that category. The developmental effects category includes general developmental effects (e.g., adverse effects including developmental damage or birth defects), whereas developmental effects that are specifically neurological in nature (e.g., adverse effects including delayed mental development or delayed or affected learning) are included in the neurological effects category. A similar approach was used to categorize beneficial health effects. PFOS, perfluorooctane sulfonate.

Figure 2 illustrates references to types of beneficial (Figure 2A) and adverse (Figure 2B) health effects in advisories and with which fish nutrients and contaminants, respectively, they are associated. There were > 4.5 times more references in advisories to adverse health effects (419 references) compared with beneficial health effects (92 references) associated with fish consumption. References to adverse non-neurological systemic effects were associated with a variety of contaminants, whereas the far more numerous references to adverse neurological effects specifically were primarily associated with mercury in fish, and to a lesser extent with seven other specific contaminants (Figure 2B). The neurological category includes cognitive effects (e.g., IQ deficits, decreased language skills, mental or physical retardation), motor effects (e.g., tremors/trembling, motor impairment, loss of coordination), nervous system effects (e.g., nervous system damage, brain damage, nerve damage), sensory effects (e.g., tingling, sensory impairment, numbness, etc.), and behavioral effects (e.g., neurobehavioral change, behavioral problems, irritability). Adverse developmental effects (e.g., delayed milestones, birth defects, developmental disabilities) were associated mostly with mercury, PCBs, and unspecified (unclear or vague) contaminant exposure.

References to beneficial health effects (Figure 2A) were made with respect to omega-3 fatty acids in fish or to unspecified (unclear or vague) fish nutrients only. References to cardiovascular benefits dominated, followed by developmental and then cognitive benefits. There were 42 references in advisories to beneficial cardiovascular health effects (e.g., heart disease prevention, heart attack prevention, lower blood pressure). Twenty-six of these references were made with respect to omega-3 fatty acids obtained through fish consumption, whereas the remaining references were not made with respect to any specific fish nutrient. There were 22 references to beneficial developmental health effects (e.g., birth defects prevention, growth benefits, cell development benefits), and half of these statements were made with respect to omega-3 fatty acids. In addition to conferring health benefits, some advisories also indicated that fish consumption provides non-health benefits such as recreation (Table 2).

### Clarity and emphasis of risks and benefits

**Figure 3:**
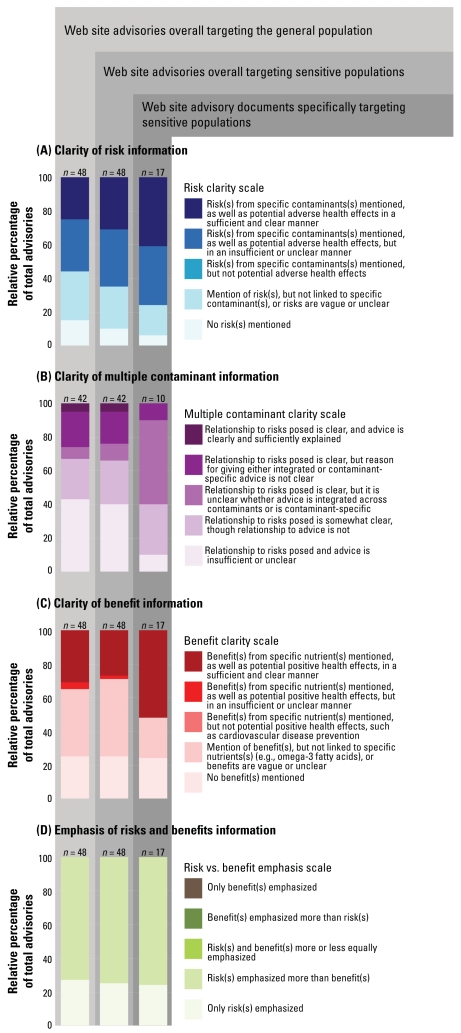
State fish consumption advisories targeted at the general population or sensitive populations: (*A*) clarity of risk information, (*B*) clarity of multiple contaminant information, (*C*) clarity of benefit information, and (*D*) emphasis of risk and benefit information. Results are shown separately for Web site documents specifically targeting sensitive populations (far right column) versus messages targeting sensitive populations both within Web pages and brochures specifically targeting sensitive populations as well as within Web pages and brochures that intermingle advice for the general population and sensitive populations (middle column).

Concerning the clarity of risk information presented in advisories, 31% (15) and 25% (12) of advisory Web sites addressed risks posed by specific contaminants and explained potential adverse health effects in a clear and sufficient manner to sensitive populations and to the general population, respectively (see Figure 3A, including scale definition). In many cases, potential risks faced by sensitive and general populations were vague, unclear, or not sufficiently explained. For example, the statement of the Georgia Department of Natural Resources, Environmental Protection Division that “your body may build up harmful levels of toxic chemicals that can affect your pregnancy and the health of your baby” (2007) is vague and is not considered sufficient, because the specific risks posed by eating fish are unclear. However, 41% (7) of advisories with documents specifically targeting sensitive populations explained risks in a clear and sufficient manner. The following statement by the Rhode Island Department of Health exemplifies clear and sufficiently explained risks: “Too much mercury can affect your baby’s brain and how your baby learns, moves, and behaves” (2007).

Five percent of the 42 advisories that addressed multiple contaminants explained the relationship between risks posed and advice in a clear and sufficient manner (see Figure 3B, including scale definition). In approximately 40% of the 42 advisories, the relationship between advice and risks posed to sensitive populations and the general population was not sufficiently or clearly explained. Among half of the 10 multiple-contaminant advisories with documents specifically targeting sensitive populations, the relationship between risks posed by multiple contaminants and advice was clear, but it was unclear whether advice was integrated across the multiple contaminants or was contaminant-specific. For example, an advisory may state that a variety of chemicals contaminate fish and may suggest certain fish to limit or avoid consuming but not make clear which suggestions are based on which chemical(s). Thus, the consumer may not be clear about the health basis driving the suggestions to limit or avoid consuming certain fish and, hence, may experience difficulty in putting the suggestions into a decision-making context.

Concerning the clarity of benefit information presented in advisories, 27% (13) and 31% (15) of advisory Web sites addressed benefits from specific nutrients and explained potential positive health effects in a clear and sufficient manner to sensitive populations and to the general population, respectively (see Figure 3C, including scale definition). In many cases, potential benefits to sensitive and general populations were vague, unclear, or not sufficiently explained. However, 52% (25) of advisories with documents specifically targeting sensitive populations explained health benefits in a clear and sufficient manner. An example by Ohio of explaining health benefits in a clear and sufficient manner is as follows: “Omega-3 fatty acids are important during fetal brain and eye development. Omega-3 fatty acids also help to prevent heart disease in adults” (2007). This statement contains specific information about health benefits associated with consuming omega-3 fatty acids that the consumer can use to make decisions about consuming fish.

In no cases were benefits emphasized equally or more than risks (see Figure 3D, including scale definition). In approximately 75% of advisories, both risks and benefits were emphasized, but risks were emphasized more than benefits to both sensitive and general populations. In the remaining cases, only risks were emphasized. An example of advice that would emphasize risks more than benefits would be advice that did state both risks and benefits of consuming fish, but devote most of the message to specific suggestions to limit or avoid consuming certain fish based on risks. The trend was similar among advisories with documents specifically targeting sensitive populations.

## General Advisory Characteristics

### Agencies issuing advisories

Table 3 illustrates categories of government agencies responsible for state fish consumption advisories. Health agencies, environmental agencies, or a combination or multiple agencies working in concert were responsible for the vast majority of advisories issued by states (Table 3). Advisories issued jointly or in a collaborative manner were issued by two or more agencies, including health, environmental, or other agencies. For example, advisories in New Mexico (2006) were issued in a collaborative manner by the Department of Health, Department of Game and Fish, State Parks, and Environment Department.

### Advisory scope

Seventy-one percent (34) of the advisory Web sites offered a combination of statewide advice or general guidance in addition to site-specific advice (Table 3). A few states [Nevada (2007), Oklahoma Department of Environmental Quality (2007), and Hawaii (2003)] offered only statewide advice or general guidance (Table 3).

### Advisory development methods

Most advisory Web sites referenced, at least to some extent, the methods used to develop advice (Table 3). Among these, 23 used what appear to be risk-based approaches (e.g., mentioned using U.S. EPA methods or risk assessment methods). Several advisories explained that estimated risks were based on a 70-year exposure duration [for instance, New Jersey (2006), Nebraska (2007), Montana (2005), and Kansas Department of Health and Environment, Department of Wildlife and Parks (2006)], whereas at least one [Georgia (2007)] used a 30-year exposure duration. Risks were based on cancer or noncancer end points or a combination of both (Table 3). At least four advisories [issued by West Virginia (2007), Ohio (2007), Kentucky Departments for Environmental Protection, Health Services, Fish and Wildlife Resources (2007), and Indiana State Department of Health, Department of Natural Resources, Department of Environmental Management (2006)] used criteria developed by the Great Lakes Task Force. The Missouri Department of Health and Senior Services (2005) advisory noted that the state is currently evaluating both U.S. EPA guidance and FDA health standards for use in developing advice, and South Dakota (2007) used FDA action levels. The Iowa Department of Natural Resources, Department of Public Health (2006) advisory states that fish consumption benefits are considered when issuing advice.

### Reference to advice issued by other entities

Twenty-seven percent (13) of advisories explicitly referenced the 2004 joint U.S. EPA/FDA fish consumption advisory and reiterated at least some, if not all, of the advice, and 23% (11) of advisories referenced advice issued by other states (Table 3). Numerous advisories recommended that sensitive populations consult their health care providers regarding fish consumption. Vermont (2005) advised residents to discuss the fish they eat with their health care providers, and West Virginia (2007) residents are advised to talk over their fish consumption concerns with their doctors. Some states, including California (2003), Washington (2007), Rhode Island (2007), and Louisiana Section of Environmental Epidemiology and Toxicology of the Department of Health and Hospitals, Department of Environmental Quality, Department of Wildlife and Fisheries, Department of Agriculture and Forestry (2006) advised advisory readers to consult their physicians regarding exposure and testing. The Mississippi Department of Environmental Quality (2007) and Rhode Island (2007) offered advice to physicians, and Maryland (2007) offered a link to U.S. EPA advice for physicians.

## Discussion

Advisories are considered voluntary recommendations regarding fish consumption and are not subject to regulation. States have primacy in protecting the public’s health from fish caught in local waters (Cunningham et al. 1994) and may choose not to issue advisories. At the time this comparative analysis was completed, Alaska and Wyoming had not issued advice. However, since that time, the Alaska Department of Health and Social Services, Division of Public Health (2007) and Wyoming Department of Health, Game and Fish Department (2007) have issued advisories. Hence, for the first time, all 50 states are issuing fish consumption advice. The following section discusses issues highlighted by the comparative analysis of fish consumption advisories issued by the 48 states assessed.

### Audience and advice

During pregnancy, women might experience a heightened awareness of and receptivity to health messages regarding potential risks to the fetus. Women of childbearing age, women who might or plan to become pregnant, and nursing women might react similarly. Because the comparative analysis revealed that all state advisories target sensitive populations, it is particularly important that these advisories offer thoughtful recommendations that consider perception of advisory content. Several advisories, including those issued by Montana (2005), New Jersey (2006), Virginia Department of Health, Public Health Toxicology (2006), Connecticut (2006), Tennessee (2007), and Pennsylvania (2007), referred to sensitive populations as “high-risk” groups, a label that sensitive populations might perceive strongly.

Those responsible for issuing advice might consider whether advisory intentions match outcomes. Many advisories suggest that women of childbearing age continue eating less-contaminated fish, but research shows these sensitive groups might, in fact, decrease overall consumption after advisories are issued (Oken et al. 2003). Are advisories designed so that sensitive populations have the information they need to continue fish consumption in a healthy way? The Alabama Department of Public Health (2006) advisory is purportedly designed to provide information so fishermen can make informed fish consumption decisions. However, sensitive populations are advised to eat no fish under advisory, but are not offered alternatives. On the other hand, some states not only offer a list of suggested fish to consume but also provide fish recipes, including Washington (2007) and Maine Department of Health and Human Services, Division of Environmental Health, Center for Disease Control and Prevention (2006). Several advisories [e.g., Michigan (2007), Ohio (2007), South Carolina (2007), and Washington (2007)] conduct surveys that aim to improve advice.

Findings suggest that sensitive populations usually receive meal size advice for specific species but usually do not receive advice on whether the size recommended relates to raw fish or to cooked fish, which differ in size. Also, sensitive populations are not likely to receive information about how advice relates to their body size in particular. This may leave sensitive populations confused and perhaps less inclined to eat fish.

### Risk and benefit messages

The comparative analysis of fish advisories showed that most advisories were based on multiple contaminants, but that few advisories, particularly those that integrated advice across more than one contaminant, described how recommendations were developed. In large part, the tools to address simultaneous contamination by multiple chemicals are likely lacking. Figure 2B shows that advisories are based on multiple contaminants that may share associations in common with increased risk of developing the same adverse health effects. This finding points to the complexity of multiple contaminant exposure and implications for human health.

The fact that approximately one-quarter of advisories do not convey that fish contain valuable nutrients is striking, because research shows that fish consumers perceive fish risks more so than benefits (Verbeke et al. 2005). Therefore, in addition to conveying risk information, advisories present opportunities to communicate health benefits to raise awareness among fish consumers.

The comparative analysis of fish consumption advisories revealed differences in how clearly risks and benefits were presented, as well as differences in clarity of messages targeting general and sensitive populations. Documents specifically targeting sensitive populations did a superior job conveying both risk and benefit messages to sensitive populations compared with advisory Web sites overall. This highlights the opportunities that these pamphlets and brochures present in educating sensitive populations.

### General advisory characteristics

The comparative analysis revealed that one-quarter of advisories are jointly issued by two or more agencies, which points to the collaborative approach many states have taken and suggests that the advisory development process may require contributions from multiple disciplines. At least one state, Minnesota Department of Environmental Quality (2007), chose to reach beyond the realm of state agencies, however, and developed advice in collaboration with dietitians.

It appears that states have tended to move away from FDA methods over time toward risk-based approaches in developing advice. According to Cunningham et al. (1994), a 1988 survey by the American Fisheries Society, requested by U.S. EPA, found that 34 states used FDA action levels to set advisories, even though FDA action levels address commercial fish and were not designed to protect those consuming recreationally caught fish. Ten states used U.S. EPA risk-based methods, and 11 states used other levels of concern to set advisories.

The analysis also revealed that numerous advisories recommended that sensitive populations consult their health care providers regarding fish consumption. The extent to which health care providers are trained and equipped to give fish consumption advice is a compelling question.

## Conclusions

Although this comparative analysis of fish consumption advisories issued by states reveals that most states do present information about benefits of consuming fish in addition to the risks, the results suggest that the message is uneven and that advisories may inadvertently cast a dim light on all fish consumption. Ideally, from a public health perspective, sensitive populations should receive clear, sufficiently explained health messages regarding fish consumption that aim to optimize both maternal and fetal health by decreasing risks and increasing benefits.

We intend in this analysis not to fault state fish consumption advisories for presenting an uneven message, but rather to suggest that the uneven message may not provide sensitive populations with the tools and information they need to make healthy, informed eating decisions regarding fish. If these state advisories are a source of decision-making information for sensitive populations, then measures to improve message clarity would be valuable. However, additional research is necessary to address the question of where these groups access fish consumption information and how these information sources affect fish consumption decisions. In the case of advisory information published online, a major factor that could impact advisory awareness is lack of Internet access, and this can be especially problematic for some of the sensitive subsistence-fishing populations that may need to hear these messages the most. Additional research is also required to evaluate how health risk and benefit information presented in the advisories compares with actual estimated health risks and benefits of fish consumption.

This study suggests that important lessons can be gained from evaluation of available state fish consumption advisories, and this should allow state agencies to collectively improve the clarity of their messages. This analysis also highlights the complexity of these messages and points to the need for additional research that can improve the public health context for our messages. One important lesson learned from this analysis is that the message to sensitive populations is uneven in terms of risks and benefits addressed, health effects mentioned, and other attributes. These differences could lead to different interpretations that do not match advisory intentions. State fish consumption advisories offered many good examples of creative approaches to communicating this information. Additional cross-agency approaches to issuing advice could prove useful in pulling together best practices. Because states have primacy in the decision to issue fish consumption advice, and contaminants of concern can vary geographically, each state needs to make distinct assessment of contaminant exposure, risks posed, and communication of those risks specific to the state. However, the U.S. EPA does provide guidance on “standardizing the approaches to evaluating risks and developing fish consumption advisories that are comparable across different jurisdictions” (U.S. EPA 2007d), although this guidance would gain from thorough consideration and incorporation of benefit communication as well. Several common denominators exist across state advisories that would gain from harmonization and coordination in particular. These include aspects of risk assessment such as dose response and hazard identification, as well as guiding principles of both risk and benefit communication and transparency of advisory development methods.

Coordination across agencies should include the development of workshops or online forums to encourage collaboration and discussion to share lessons learned and to move toward harmonizing approaches, including the development of best practices for specific media (e.g., Web-based, print) to communicate benefits. An additional way to help provide a more complete picture of risks and benefits is to develop standard metrics for describing the benefits of omega-3 fatty acids across fish, for example, as well as standard metrics for describing risks of contaminants. Attempts have been made to develop standard metrics, and we think consumers would benefit from the development of a profiling model that expresses a score for fish combining both toxicological and nutritional information to help guide consumers toward species that would confer fewer potential risks and greater benefits (Drewnowski and Fulgoni 2008; Scherer et al. 2008).

## Figures and Tables

**Table 1:** Audience and advice attributes of the 48 state fish consumption advisory Web sites assessed.

Attribute	No. (%)
Sensitive populations targeted	
Pregnant women	43 (89.6)
Women of childbearing age	28 (58.3)
Women planning to become pregnant	20 (41.7)
Women who might become pregnant	27 (56.3)
Children	48 (100.0)
High-end fish consumers	6 (12.5)
People with certain health conditions	3 (6.3)
Languages available	
Spanish	18 (37.5)
Vietnamese	5 (10.4)
Chinese	4 (8.3)
Korean	3 (6.3)
Hmong	3 (6.3)
Russian	3 (6.3)
Khmer	2 (4.2)
Laotian	2 (4.2)
Cambodian	2 (4.2)
Serbo-Croatian	1 (2.1)
French	1 (2.1)
Haitian Creole	1 (2.1)
Portuguese	1 (2.1)
Metrics of advice: meal frequency and size	
Offer meal frequency advice	47 (97.7)
Recommend no. of meals per week	38 (79.2)
Recommend no. of meals per month	33 (68.8)
Recommend no. of meals per year	7 (14.6)
Offer species-specific advice	46 (95.8)
Offer meal size advice	36 (12.5)
Offer meal size advice for adults	23 (47.9)
Offer meal size advice for children	18 (37.5)
Offer meal advice based on body weight	10 (20.8)
Advice based on fish length in inches	30 (62.5)
Advice based on size of fish caught in pounds	4 (8.3)
Meal size advice based on uncooked fish portions	9 (18.8)
Meal size advice based on cooked fish portions	3 (6.3)
Meal size advice based on both cooked and uncooked fish portions	4 (8.3)
Cooking and preparation advice	
Offer cooking and preparation advice	28 (58.3)
Provide a fish preparation graphic	21 (43.8)

**Table 2:** Contaminants, nutrients, and non-health effects presented in the 48 state fish consumption advisory Web sites assessed.

Attribute	No. (%)
Contaminants addressed	
Single contaminant only	6 (12.5)
Multiple contaminants	42 (87.5)
Multiple contaminants, and advice is contaminant-specific^a^ only	9 (18.8)
Multiple contaminants, and at least some advice is integrated^b^	29 (60.4)
Integrated advice, but no explanation of how developed	22 (45.8)
Some explanation of integrated advice development	7 (14.6)
Mentions detection of or risks posed by chemicals not under advisory	13 (27.1)
Nutritional aspects addressed	
Protein source	37 (77.1)
Omega-3 fatty acid source^c^	22 (45.8)
Vitamin source	16 (33.3)
Mineral source	16 (33.3)
Nutritious/source of nutrients	12 (25.0)
Low in cholesterol	5 (10.4)
Low in calories	3 (6.3)
Low in sodium	2 (4.2)
Low in fat	23 (47.9)
Low in saturated fat specifically	16 (33.3)
References other protein sources	11 (22.9)
Non-health benefits addressed	
Recreation source	17 (35.4)
Provide food/supports a subsistence lifestyle	6 (12.5)
Cultural, spiritual, or traditional relevance	2 (4.2)
Economic importance	4 (8.3)

a For example, advice for one water body was based on mercury risks whereas another was based on PCB risks.

b For example, advice for a particular water body was based on risks from both mercury and PCBs together.

c Or beneficial or good oils or fats, which likely refer to omega-3 fatty acids, and which are included in that category.

**Table 3:** General characteristics of the 48 state fish consumption advisory Web sites assessed.

Attribute	No. (%)
Issuing agency	
Health^a^	24 (50.0)
Environmental^b^	7 (14.6)
Health and environment^c^	2 (4.2)
Jointly issued by two agencies	5 (10.4)
Jointly issued by three agencies	6 (12.5)
Jointly issued by four agencies	1 (2.1)
Other^d^	3 (6.3)
Scope	
Statewide/general guidance only	3 (6.3)
Site-specific advice only^e^	11 (22.9)
Combination of statewide/general guidance and site-specific advice	34 (70.8)
Advice on locally caught fish only	18 (37.5)
Advice on both locally and commercially caught fish	30 (62.5)
Advice on finfish species only	18 (37.5)
Advice on both finfish and shellfish species^f^	30 (62.5)
Advisory development methods	
Reference advisory development methods	28 (58.3)
Use what appear to be risk-based methods	23 (47.9)
Estimate cancer risk	9 (18.8)
Estimate noncancer risk	7 (14.6)
Estimate both cancer and noncancer risk	6 (12.5)
Reference advice issued by other entities	
Reference and reiterate the 2004 joint U.S. EPA/FDA advice	13 (27.1)
Reference advice issued by other states	11 (22.9)

a Departments and divisions of health, health and senior/human services, health and hospitals, public health, community health, and environmental health.

b Environmental protection, conservation, management, quality, and services, as well as departments of the environment or natural resources.

c The unique so-called health and environment agency does not fall into either the health or environment categories.

d Agencies such as game and fish commissions and food or seafood quality divisions that did not fit into other categories well.

e Advice pertinent to particular water bodies, counties, etc.

f Shellfish include mollusks (e.g., clams, oysters, octopus, squid, snails) and crustaceans (e.g., crab, lobster, crayfish).
